# Comparative study of percutaneous endoscopic lumbar decompression and traditional revision surgery in the treatment of symptomatic adjacent segment degeneration

**DOI:** 10.1186/s12893-024-02470-8

**Published:** 2024-06-07

**Authors:** Jianwei Guo, Changlin Lv, Tianyu Bai, Guanghui Li, Xiangli Ji, Kai Zhu, Guoqing Zhang, Xuexiao Ma, Chong Sun

**Affiliations:** 1 Department of Spinal Surgery, The Affiliated Hospital of Qingdao University, 16 Jiangsu Road, Qingdao, Shandong Province 266003 People’s Republic of China; 2 Department of Intensive Care Unit, Qilu Hospital of Shandong University (Qingdao), 758 Hefei Road, Qingdao, Shandong Province 266035 People’s Republic of China; 3 Department of Orthopedics, The Affiliated Hospital of Qingdao University, 16 Jiangsu Road, Qingdao, Shandong Province 266003 People’s Republic of China

**Keywords:** Adjacent segment degeneration, Revision surgery, Percutaneous lumbar endoscopic discectomy, Comparative study

## Abstract

**Objective:**

The objective of this study is to evaluate and compare the surgical outcomes and complications of Percutaneous Endoscopic Lumbar Decompression (PELD) and traditional revision surgery in treating symptomatic Adjacent Segment Degeneration (ASD). This comparison aims to delineate the advantages and disadvantages of these methods, assisting spine surgeons in making informed surgical decisions.

**Methods:**

66 patients with symptomatic ASD who failed conservative treatment for more than 1 month and received repeated lumbar surgery were retrospectively collected in the study from January 2015 to November 2018, with the average age of 65.86 ± 11.04 years old. According to the type of surgery they received, all the patients were divided in 2 groups, including 32 patients replaced the prior rod in Group A and 34 patients received PELD at the adjacent level in Group B. Patients were followed up routinely and received clinical and radiological evaluation at 3, 6, 12 months and yearly postoperatively. Complications and hospital costs were recorded through chart reviews.

**Results:**

The majority of patients experienced positive surgical outcomes. However, three cases encountered complications. Notably, Group B patients demonstrated superior pain relief and improved postoperative functional scores throughout the follow-up period, alongside reduced hospital costs (*P* < 0.05). Additionally, significant reductions in average operative time, blood loss, and hospital stay were observed in Group B (*P* < 0.05). Notwithstanding these benefits, three patients in Group B experienced disc re-herniation and underwent subsequent revision surgeries.

**Conclusions:**

While PELD offers several advantages over traditional revision surgery, such as reduced operative time, blood loss, and hospital stay, it also presents a higher likelihood of requiring subsequent revision surgeries. Future studies involving a larger cohort and extended follow-up periods are essential to fully assess the relative benefits and drawbacks of these surgical approaches for ASD.

## Introduction

Posterior laminectomy and fusion with pedicle screws is a prevalent treatment modality for Lumbar Degenerative Diseases (LDD) [1, 2]. Due to solid fixation caused by the internal instruments at surgical segments, the mechanical stress at the adjacent segments is increased, which may accelerate the degeneration rate of adjacent segments [3–5]. The recurrence of symptoms associated with the degeneration at the adjacent segment will occur after a symptom-free period. Adjacent segment degeneration (ASD) is defined as the radiological changes of the intervertebral discs adjacent to the pre-surgical spinal level, regardless of the presence of symptoms [1, 6, 7]. According to earlier reports, the occurrence of ASD after lumbar spinal fusion surgery have been observed in 36–84% patients at the 5-year follow-up and the incidence of symptomatic ASD requiring reoperation ranges from 5.2 to 18.5% [8], highlighting it as a significant concern among spinal surgeons.

In cases where ASD becomes symptomatic and conservative management fails, various surgical interventions are considered [9]. Different methods have been used to deal with this problem, such as open posterior laminectomy with extension of the instrumented fusion [10], anterior lumbar interbody fusion (ALIF) [11], oblique lumbar interbody fusion (OLIF) [12], extreme lateral interbody fusion (XLIF) [13], and endoscopic surgery [14]. Despite its known effectiveness, the traditional open posterior laminectomy and extension surgery, often favored for its familiarity, necessitates resection at primary surgical sites and rod removal [9]. This approach can lead to secondary damage to paraspinal muscles, potentially inducing chronic back pain, muscle weakness, and long-term functional disability [15, 16]. Additionally, excision of previous surgical scars may increase the risk of dural tear and extensive surgical trauma to the paraspinal muscle.

In contrast, Percutaneous Endoscopic Lumbar Decompression (PELD) has gained traction in recent years as a viable, minimally invasive alternative for treating lumbar herniation and spinal stenosis [17]. PELD requires only a minimal incision, mitigating damage to facet joints and posterior ligaments while preserving the stability of the surgical vertebral segment [18, 19]. Given its minimal invasiveness and reduced tissue disruption, PELD is hypothesized to be effective for treating symptomatic ASD resulting from spinal stenosis or herniated discs, even in the absence of overt segmental instability. Although PELD’s application in ASD treatment has been documented, comparative studies on its efficacy and complication rates against traditional revision surgeries remain scant. This study aims to evaluate and contrast the surgical outcomes and complications of PELD and traditional revision surgery, thereby aiding spine surgeons in making informed decisions regarding ASD management.

## Materials and methods

This study was a clinical retrospective study and approved by the Medical Ethics Committee of the Affiliated Hospital of Qingdao University. Informed consents were obtained from all the individual enrolled in the study. The study cohort comprised patients with symptomatic Adjacent Segment Degeneration (ASD) who had failed conservative treatment for over one month and underwent repeat lumbar surgery from January 2015 to November 2018. Patient inclusion criteria were (1) Previous open lumbar surgery and fusion with pedicle screws, (2) Symptoms attributed to herniated discs or lumbar stenosis at the adjacent level, (3) Failure of conservative treatment for at least one month, (4) Radiological absence of dynamic instability at the ASD level, (5) Minimum of one year of follow-up. Patients with active infection, malignancy, acute trauma, serious neurological deficit, spinal instability, or follow-up time less than 1 year were excluded from this study.

Diagnostic assessments, including lumbar X-ray, computed tomography (CT), and magnetic resonance imaging (MRI), were conducted to identify herniated discs or canal stenosis at the adjacent levels prior to the revision surgeries. Patients were stratified into two groups based on the preferences and expertise of senior surgeons. Group A consisted of patients who underwent laminectomy and extension fusion surgery at the adjacent level with replacement of the longer rod. Group B included patients who underwent Percutaneous Endoscopic Lumbar Decompression (PELD) at the adjacent level without fixation. Data on general characteristics such as age, sex, underlying diseases, ASD level, time intervals between the operations, operative time, intraoperative blood loss, hospital stay, hospital costs, and complications were systematically recorded through chart reviews.

### Surgical procedures

Prior to surgery, all participants were thoroughly informed about the procedural steps and provided informed consent for the surgical interventions.

**Fig. 1:**
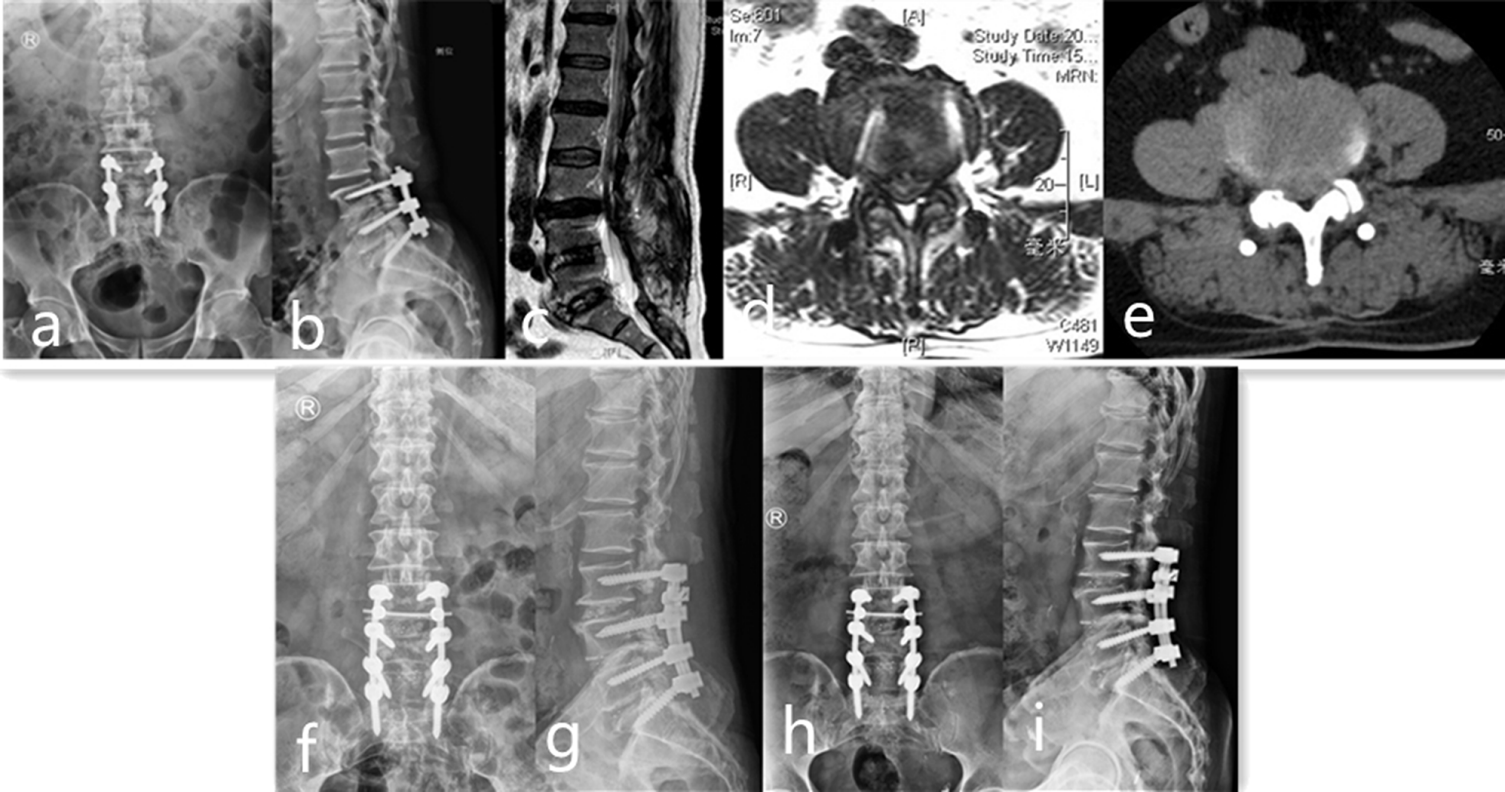
A 65-year-old female was diagnosed as symptomatic ASD at the L3/4 level. He was performed with posterior decompression and internal fixation at L4-S1 due to disc herniation 2 years ago. **a, b** Preoperative X-ray showed posterior fixation with an intervertebral cage at L4/5 and mild posterior displacement at L3/4. **c, d, e, f** Preoperative MRI and CT showed disc herniation at L3/4, which compressed dura sac and L4 nerve root. **f, g **Postoperative X-ray showed posterior decompression and fixation surgery was performed at L3/4 and longer rods were used to connect the ASD level with the primary surgical sites. **h, i** Postoperative X-ray at 1-year follow-up showed good results were achieved and no instrumentation breakage and displacement were found

Group A: Patients underwent laminectomy and extension fusion surgery at the adjacent level, where prior rods were replaced with longer rods to accommodate the extended fusion (Fig. 1). Postoperatively, patients were allowed ambulation once the drainage tubes were removed. They were required to wear lumbar braces for three months to support the surgical site during the initial healing phase.

**Fig. 2:**
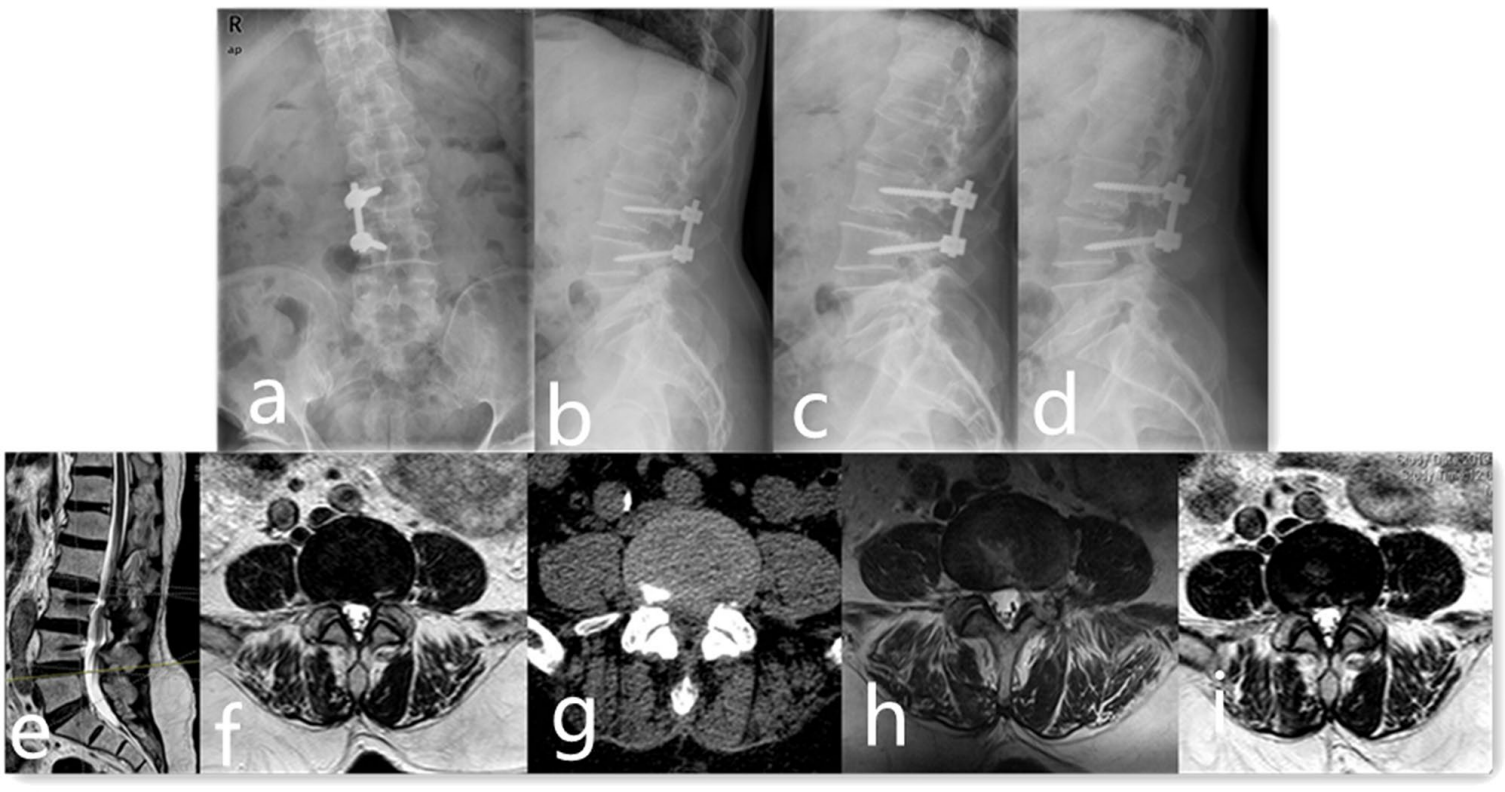
A 66-year-old female was diagnosed as symptomatic ASD at the L4/5 level. He was performed with posterior decompression and internal fixation at L3-4 due to disc herniation 3 years ago. **a, b, c, d** Preoperative anterior-posterior, lateral and dynamic lumbar X-ray showed posterior fixation with an intervertebral cage at L3/4 and no instability was found at L4/5. **e, f, g** Preoperative MRI and CT showed disc herniation at L4/5, which compressed L5 left nerve root. **h** Postoperative MRI showed percutaneous transforaminal endoscopic lumbar surgery was performed at the left side of L4/5 and protruded disc was removed. i Postoperative MRI at 1-year follow-up showed good results were achieved and no disc herniation was found

Group B: Patients received Percutaneous Endoscopic Lumbar Decompression (PELD) at the adjacent level without any fixation (Fig. 2). The procedure was conducted using either a transforaminal or interlaminar approach, depending on the specific anatomical and pathological requirements of each case. This method allowed for targeted decompression with minimal disruption to surrounding structures. These patients were permitted to walk one day post-operation, with the aid of a lumbar brace for one month to ensure adequate support and stability as they resumed mobility.

### Clinical and radiological evaluation

Patients were systematically followed up with scheduled clinical and radiological evaluations at 3, 6, and 12 months postoperatively, and annually thereafter. Several standardized tools were used to evaluate the clinical outcome, such as visual analog scale (VAS) for low-back and leg pain, Oswestry disability index (ODI) for functional disability, and modified Macnab criteria for patients’ satisfaction. Visual Analog Scale (VAS) was used to measure the intensity of low-back and leg pain, providing a subjective measure of pain severity. Oswestry Disability Index (ODI) was used to evaluate functional disability, helping quantify the patient’s ability to manage everyday life activities. Modified MacNab Criteria was employed to assess patient satisfaction with the outcomes of the surgery. Additionally, any complications encountered during the follow-up period and associated hospital costs were meticulously recorded through chart reviews, allowing for a comprehensive evaluation of the surgical interventions’ efficacy and economic impact.

### Statistical analysis

All statistical analyses were conducted using SPSS software (version 17.0, Chicago, USA). Continuous variables, such as operative time, blood loss, and hospital stay, were expressed as mean ± standard deviation (SD) and compared using paired t-tests. Categorical variables, including complication rates and patient satisfaction levels, were analyzed using the Mann–Whitney U test, Fisher exact test, and chi-square test as appropriate. A P value of less than 0.05 was considered statistically significant, indicating a meaningful difference between the groups under comparison.

## Results

In this study, 66 patients met the inclusion and exclusion criteria, including 30 female and 36 male patients, with the average age of 65.86 ± 11.04 years old. Patients were categorized into two groups based on the surgical procedure they underwent: Group A (32 patients) where the prior rod was replaced with a longer rod alongside laminectomy at the adjacent level, and Group B (34 patients) which involved Percutaneous Endoscopic Lumbar Decompression (PELD) at the adjacent level without fixation. Within Group B, 20 patients underwent the interlaminar approach and 14 patients underwent the rransforaminal approach. The baseline characteristics such as age, sex, time interval between initial and revision surgeries, location of ASD, and preoperative scores for Visual Analog Scale (VAS) for back and leg pain, as well as Oswestry Disability Index (ODI), were comparable between the groups, with no significant differences noted (Table 1).

**Table 1:** Comparison of demographic and surgical data among Groups

	Group A	Group B	*P* value
Number of patients	32	34	
Sex			0.831
Female	17	13	
Male	15	21	
Age (Years)	63.03 ± 9.39	62.88 ± 11.76	0.955
No. of fused levels at index surgery	1.75 ± 0.57	1.76 ± 1.35	0.955
Interval between index surgery and revision surgery (months)	52.71 ± 44.74	75.88 ± 52.57	0.059
Location of ASD levels			0.895
L1/2	1	2	
L2/3	8	2	
L3/4	14	10	
L4/5	4	10	
L5/S1	5	10	
Underlying diseases			0.421
Hypertension	8	5	
Diabetes	7	3	
Coronary heart diseases	3	2	
Other diesease	2	2	
Surgical time (minutes)	228.19 ± 83.33	95.35 ± 38.27	0.000*
Estimate blood loss (mL)	360.00 ± 168.66	25.44 ± 6.30	0.000*
Mean hospital stay (days)	13.34 ± 6.96	5.18 ± 1.98	0.000*
Complications	0	3	0.072
Length of outpatient follow up (months)	37.78 ± 22.33	34.71 ± 19.45	0.552
Total hospital cost, USD	6166.59 ± 2451.01	3315.01 ± 154.27	0.000*
Modified Macnab satisfaction(Excellent-good, %)	87.50%	88.24%	0.933

*P value < 0.05

The majority of patients reported favorable surgical outcomes, although three cases encountered complications. Detailed clinical outcomes, depicted in Table 2, show significant postoperative relief in back and leg pain, alongside improvements in functional outcomes during the follow-up period. Notably, Group B patients exhibited superior pain relief and functional scores, achieving these results with significantly lower hospital costs (*P* < 0.05) (Fig. 1). Additionally, Group B experienced reductions in average operative time, blood loss, and hospital stay, all reaching statistical significance (*P* < 0.05).

**Table 2:** The comparison of clinical outcomes among Groups

	Group A	Group B	*P* value
VAS for lumbar pain			
Preoperative	5.06 ± 1.29	4.94 ± 1.59	0.736
3-month follow-up	3.84 ± 1.08	2.85 ± 0.86	0.000*
6-month follow-up	3.31 ± 0.93	2.53 ± 0.51	0.000*
12-month follow-up	2.91 ± 0.82	2.21 ± 0.81	0.001*
Final follow-up	1.88 ± 0.83	2.00 ± 0.82	0.540
VAS for leg pain			
Preoperative	6.75 ± 1.08	7.21 ± 1.20	0.110
3-month follow-up	3.22 ± 0.61	2.53 ± 0.56	0.000*
6-month follow-up	2.72 ± 0.73	2.50 ± 0.71	0.220
12-month follow-up	2.18 ± 0.74	2.09 ± 0.67	0.568
Final follow-up	1.69 ± 0.86	1.59 ± 1.02	0.671
ODI			
Preoperative	64.63 ± 6.97	63.76 ± 5.09	0.567
3-month follow-up	19.47 ± 5.65	15.76 ± 3.83	0.003*
6-month follow-up	19.97 ± 4.50	15.29 ± 2.34	0.000*
12-month follow-up	15.75 ± 3.72	13.71 ± 1.71	0.002*
Final follow-up	14.09 ± 3.76	10.76 ± 2.36	0.000*

*P value < 0.05 VAS: visual analog scale, ODI: Oswestry disability

The study observed no serious neurological complications, infections, or rod breakage in any of the patients across both groups. Notably, three patients from Group B experienced a recurrence of disc herniation, necessitating secondary surgeries at varied follow-up intervals—specifically at 3 months, 6 months, and 2 years post-initial surgery. Among the three patients, 2 patients received transforaminal approach and one patient underwent posterior decompression surgery with instruments. In terms of structural stability, no dynamic instability was detected in either Group A or Group B during the final follow-up. Additionally, assessments showed no evidence of cage subsidence, screw loosening, or rod breakage in Group A.

## Discussion

Adjacent Segment Degeneration (ASD) is an increasingly recognized complication following lumbar fusion surgery, with biomechanical alterations at the levels adjacent to the fixed segments contributing significantly to this phenomenon [1, 20]. A biomechanical study has demonstrated that stress on L3/4 vertebral endplate and intervertebral discs on flexion/extension moment increased after fusion at the L4/5 level [21]. A cadaveric experiment revealed that L2/3 intradiscal pressure on flexion/extension stress increased 45% in the cadaveric L3/4 fixation model [22]. Although the causes of ASD may be multifactorial, the biomechanical changes at the adjacent level after fusion surgery will accelerate the degeneration of intervertebral discs, which may cause the radiographic changes in the intervertebral discs at the adjacent level and even become symptomatic.

When conservative treatments for symptomatic ASD fail, a range of surgical options are considered. These include traditional methods like posterior decompression and extended fusion, and advanced techniques such as anterior lumbar interbody fusion (ALIF) [23], extreme lateral interbody fusion (XLIF) [24], oblique lumbar interbody fusion (OLIF) [12, 25–27], and endoscopic surgery [28]. Although ALIF/XLIF/OLIF has been recommended by some experts for the treatment of symptomatic ASD with the advantages of less paraspinal muscle injury, low risk of operative dural tear, and less disturbance to nerve roots or cauda equina, the high costs of implants and limited familiarity with some of these advanced techniques restrict their widespread adoption [9, 25, 26].

This study highlights the efficacy of Percutaneous Endoscopic Lumbar Decompression (PELD) over traditional revision surgery, demonstrating significant advantages in terms of reduced blood loss, operative time, hospital stay, and overall costs, all of which bear statistical significance. PELD can not only remove the protruded disc and hyperplastic ligaments and articular processes to achieve good surgical outcome, but also have smaller incision and preserve paraspinal muscles and vertebral elements, which may decrease the risk of postoperative back pain. Besides, due to less damage to vertebral elements and magnification of endoscope, PELD has less incidence of dura sac injury.

However, PELD seems to have relatively higher recurrence rate. According to Telfeian’ report, 9 patients with ASDs received transforaminal endoscopic surgery and 3 patients received revision surgeries within 2 years follow-up [28]. Gu et al. [29] reported that 25 elderly ASD patients were performed with transforaminal endoscopic discectomy (PTED) and 84.0% of the patients (21/25) achieved excellent or good clinical outcomes. Only 3 patients developed complications, including 1 case of dural laceration, 1 case of postoperative dysesthesia and 1 case of recurrence. In our study, 34 cases with ASDs achieved 65.93% improvement in leg pain and 75.81% improvement in ODI scores postoperative, and only 3 cases received second revision surgery due to disc re-herniation during the follow-up, which was in consistent with earlier reports [28–30]. Compared with the interlaminar approach, the transforaminal approach seems to have a higher recurrence rate to treat symptomatic ASDs.

This study has several limitations. This study’s a retrospective, non-randomized design introduces potential biases, including variability in ASD type, prior surgical procedures, and surgeon preferences. Additionally, the relatively small sample size and the short duration of follow-up limit the generalizability of the findings. Future studies with a larger sample size and longer follow-up periods are necessary to validate these results and potentially adjust treatment protocols based on long-term outcomes.

## Conclusion

This study compared two distinct surgical approaches for treating Adjacent Segment Degeneration (ASD): traditional revision surgery and Percutaneous Endoscopic Lumbar Decompression (PELD). The findings indicate that PELD offers several benefits over traditional surgery, including reduced operative time, less blood loss, shorter hospital stays, decreased medical costs, and improved postoperative outcomes in terms of back pain relief and patient satisfaction. However, it also appears that PELD is associated with a higher likelihood of requiring additional revision surgeries.

The promising results observed with PELD highlight its potential as a viable alternative to traditional methods, particularly for patients prioritizing quicker recovery and reduced procedural impact. Nonetheless, the increased revision rate observed with PELD underscores the need for careful patient selection and postoperative management. To further elucidate the long-term benefits and limitations of these surgical options, future research should include a larger patient cohort and extended follow-up periods, enabling a more comprehensive evaluation of the techniques in the context of ASD management.

## Data Availability

The original data can be achieved by contacting the first author or corresponding author.

## Abbreviations

ASD: Adjacent Segment Degeneration
ASDs: Symptomatic Adjacent Segment Degeneration
VAS: Visual Analog Scale
ODI: Oswestry Disability Index
ALIF: Anterior Lumbar Interbody Fusion
OLIF: Oblique Lumbar Interbody Fusion
XLIF: Extreme Lateral Interbody Fusion
PELD: Percutaneous Endoscopic Lumbar Decompression
CT: Computed Tomography (CT)
MRI: Magnetic Resonance Imaging
SD: Standard Deviation
PELD: Percutaneous Endoscopic Lumbar Discectomy
